# Participation in Decision Making as a Property of Complex Adaptive Systems: Developing and Testing a Measure

**DOI:** 10.1155/2013/706842

**Published:** 2013-11-21

**Authors:** Ruth A. Anderson, Donde Plowman, Kirsten Corazzini, Pi-Ching Hsieh, Hui Fang Su, Lawrence R. Landerman, Reuben R. McDaniel

**Affiliations:** ^1^Duke University School of Nursing, DUMC Box 3322, Durham, NC 27710, USA; ^2^The University of Nebraska-Lincoln, College of Business Administration, Lincoln, NE 68508, USA; ^3^National Taipei University of Nursing and Health Sciences, Health and Nursing Service Administration Department, Beitou District, Taipei City 112, Taiwan; ^4^Duke University, Center for the Study Aging and Human Development, Durham, NC 27710, USA; ^5^The University of Texas at Austin, McCombs School of Business, Austin, TX 78705, USA

## Abstract

*Objectives.* To (1) describe participation in decision-making as a systems-level property of complex adaptive systems and (2) present empirical evidence of reliability and validity of a corresponding measure. *Method.* Study 1 was a mail survey of a single respondent (administrators or directors of nursing) in each of 197 nursing homes. Study 2 was a field study using random, proportionally stratified sampling procedure that included 195 organizations with 3,968 respondents. *Analysis.* In Study 1, we analyzed the data to reduce the number of scale items and establish initial reliability and validity. In Study 2, we strengthened the psychometric test using a large sample. *Results.* Results demonstrated validity and reliability of the participation in decision-making instrument (PDMI) while measuring participation of workers in two distinct job categories (RNs and CNAs). We established reliability at the organizational level aggregated items scores. We established validity of the multidimensional properties using convergent and discriminant validity and confirmatory factor analysis. *Conclusions.* Participation in decision making, when modeled as a systems-level property of organization, has multiple dimensions and is more complex than is being traditionally measured. Managers can use this model to form decision teams that maximize the depth and breadth of expertise needed and to foster connection among them.

## 1. Introduction

Participation in decision making (PDM) by workers, including all types of nurses, is recognized internationally as an important aspect of effective management in nursing and healthcare [1–5]. Prior researchers, however, have noted deficiencies in the conceptualization of PDM [5, 6]. Two factors, little consensus as to its specific meaning [7] and various definitions [8], contribute to inconsistent findings regarding outcomes of participation. These deficiencies may stem from a simplistic view of PDM [4, 5, 9–12], typically measured at the individual level, ignoring systems-level properties.

Complexity science suggests that organizations are complex adaptive systems [13, 14] and as such PDM is an emerging property of the system. Emerging properties are generated by local interactions [15] and are particularly important for organizational adaption in complex, chaotic environments, such as the health care environment [16]. Participation emerges as nursing staff and managers of varying expertise and values interact at the local level through a variety of means (e.g., chance encounters, informal meetings, and committee structures) in making formal and informal decisions [5, 11, 12, 17–19]. The purpose of this paper is to (1) describe a measure of PDM as a systems-level property of complex adaptive systems and (2) present empirical evidence of the reliability and validity of the measure using data from two independent studies.

Emerging properties of complex adaptive systems [20], such as participation, are influenced by the quality of connections among organizational agents [11, 17, 21] and connections among agents define the future state of systems [22]. Thus, we define participation as the use of existing or new organizational connections and relationships for exchanging information in decision making. As a systems-level property, PDM is important because available information is expanded through people who are sources and processors of information and who, through varied interactions, are capable of developing multiple interpretations of information. As such, PDM enables richer understandings of events and actions as they unfold thereby enabling more effective self-organization and sense making [5, 11, 17, 18, 23, 24]. Specifically, organizations in which there are a large number of ties or connections are more capable of variety in their behavior and that leads to adaptability [11, 19, 25]. Thus, we model PDM in terms of interrelationships and connections among organizational agents. Our model differs from traditional conceptualizations of PDM in three ways.

First, PDM is normally modeled as an individual-level phenomenon whereas we reveal its systems-level properties. Observations of PDM traditionally focus on the extent to which individual workers feel they get to share with their supervisors in making decisions that affect them [1, 26, 27]. While individuals' perceptions of their own participation are important aspects of work environments, these measures do not consider that PDM is a systems-level property.

Second, PDM is most often viewed as a simple one-dimensional phenomenon whereas we model it as multidimensional, adding several dimensions to the decision processes offered by Black and Gregersen [10] and others [5, 19]. Researchers have treated PDM as a unitary concept, such as “shared influence,” that is largely captured with single-dimension questions [28]. For example, researchers ask respondents how frequently they participate in decisions or whether they are members of a decision making group, and these are viewed as indicators of the total amount of PDM [10]. Complexity theory suggests that PDM is a way that organizations complicate themselves [5, 9, 29] matching internal variety with external variety [30]. When agents in complex adaptive systems participate in decision making, a seemingly simple thing, their patterns of interaction may result in complicated phenomena at the systems level [9]. This complicated internal landscape has multiple dimensions: participation can be formal or informal [5, 28], can occur in one or several of the decision making stages [10, 19], can happen through a variety of organizational mechanisms, and can include few or many individuals [31].

Third, PDM is often treated as a strategy for affecting worker behavior while we see it as a fundamental system characteristic that affects organizational behavior. We depart from the traditional view that PDM is a strategy for gaining acceptance and commitment of workers in hopes of increased effort [32]. Because it creates interactions at the local level, PDM emerges as a systems-level property that is inherent to complex adaptive systems [33]. PDM is an important concept for nursing systems management research because it enhances the ability of organizations to gather information from their environments and about their own behavior and use this information in adapting to changes in both [34]. Thus, PDM as a property of organizations helps them to change while also exhibiting coherent behavior [11, 35]. Interactions among agents have a multiplier effect and a recycling effect, both of which serve to change the quality and quantity of information resources available to organizations [36]. When, through PDM, information is allowed to flow freely, the ability of organizations to self-organize, make sense of the world, and make meaningful decisions is enhanced [9] and they are more likely to achieve their goals.

*A Measure That Accounts for Context and Multiple Dimensions.* Because different decisions required different types of information and interpretations, PDM requires use of multiple connections and relationships [29, 37], so that people with varying knowledge and skills can exchange the information for sense making and decision making that is required in the particular decision situation [7]. This suggests that a measure of PDM must include the decision context. Thus organizational members will participate in some decisions but not others, depending on the relevance of their particular expertise related to the decision context [38, 39]. Decision context is important in measuring PDM in a health care setting because of the multiple professionals and quasiprofessionals involved in health care delivery. *Thus, a valid measure of PDM will differentiate between PDM of agents with distinctly different professional expertise.*

Decisions made in organizations can be classified as either strategic or tactical [40]. Strategic decisions relate to choices about activities designed to match an organization's mission and goals with changing conditions and opportunities in the environment. Two categories of strategic decisions are common (Table 5): (a) operations strategic decisions concern the transformation of organizational inputs into final form, and (b) marketing strategic decisions concern making services and products known and motivating consumers to use them. Tactical decisions are the day-to-day decisions that ensure that the work of organizations is accomplished in a manner that meets organizational goals. Two categories of tactical decisions are common: (a) core-functions decisions concern the application of technologies for carrying out operations decisions (e.g., patient care issues), and (b) support-functions decisions concern the activities which support effective accomplishment of core functions (e.g., scheduling issues). *Thus, PDM will vary depending on the content of a decision; for example, workers that are closer to patient care would be expected to possess the most relevant information about tactical level decisions.*

**Figure 1:**
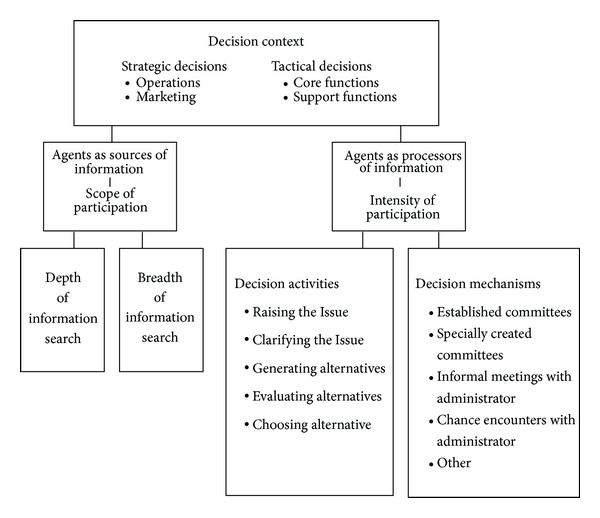
Components of the participation in decision-making instrument. Participation in decision making has two major dimensions, Scope and Intensity. Scope is comprised of breadth and depth of information brought by participants. Intensity is comprised of decision activities and mechanisms through which decision participants process information. Decision making takes place within the context of the decisions to be made, which for organizational decision making are categorized as strategic (operations or marketing) and tactical (core functions and support functions).

PDM as a system-level property in organizations has two major dimensions: *Scope*—how widely the organization searches for information from its members in terms of *depth* and *breadth* of content and *Intensity*—how extensively agents connect and interact across decision *activities* and *mechanisms* (Figure 1).

*Scope.* In complex adaptive systems, managers enable appropriate connections and interactions by varying the agents involved according to their depth of knowledge for a decision context [41]. In addition, managers enhance the value of connections and interactions by varying the agents involved according to the breadth of knowledge they bring to the decision context. These complex social structures often are tied to occupational, technical, or professional groups. A wider range of PDM brings together differing agents who bring different interpretations to the data.

*Intensity.* Organizational decision making varies according to the points in the decision process where agents connect to the decision activities and the mechanisms through which agents interact. Providing access to various decision activities increases the number of points of connection and expands organizational capacity to process information and create new meanings [33]. The capacity of organizations to deploy information increases when multiple formal and informal mechanisms for interaction are used. *A valid measure of PDM as a systems-level property will account for both dimensions of Scope and Intensity.*

*Development of the PDMI.* We operationalized the model of PDM (Figure 1) by the participation in decision-making instrument (PDMI). The items in the instrument correspond with complexity theory previously discussed and the literature on organizational designs that enhance information processing and on the nature of decision processes as described in more detail by Ashmos and McDaniel [41, 42]. The items reference the target group who's participation is being measured; in our examples in the paper the target groups are registered nurses (RNs) and certified nurse assistants (CNAs). The items were developed in a Delphi study including 20 experts who identified and evaluated the decision statements, achieving between 90% and 100% agreement that the decisions belong to the content categories shown in Table 5.

*Items Measuring Scope and Intensity Dimensions of Participation in Decision Making.* The PDMI measured the dimension of Scope through indicators of “*depth*” and “*breadth*” (Figure 1) as shown in the example in Figure 3. Depth of PDM signifies the degree to which a target group, (e.g., RNs, or CNAs) plays a major role in the decision process and was measured with the item, “Of all of the people involved in this decision, what proportion would be RNs [or insert target group identifier]?” Response options ranged from 1 (very low) to 10 (very high). Breadth of PDM signifies the degree to which a variety of view points of the target group's view points are represented in the decision process and was measured with the item, “Of all of the RNs [or insert target group identifier] in this organization, what percentage would be involved in this decision?” Response options ranged from 1 (very low) to 10 (very high).

The dimension of Intensity was measured using indicators of “decision *activities*” and “*mechanisms*” (Figure 1). Decision *activities* are points in decision processes where agents connect to the decisions and were measured with the item, “Decision activities in which RNs [or insert target group identifier] would be involved: (check all that apply).” Response options included raising the issue, clarifying the problem, generating alternatives, evaluating alternatives, and choosing alternatives. Decision *mechanisms* are the ways in which interactions take place and were measured with the item, “Mechanisms through which RNs [or insert target group identifier] would be involved: (check all that apply).” Response options included established committees, specially created committees, informal meetings with administrators, chance encounters with administrators, or “other” (write-in by respondent). During analysis, the Intensity items were recoded to a 1 to 10 scale. These four items were repeated as shown in Figure 3 for each of the context specific decision (Table 5).

*Identifying Decision Context.* The four PDMI items described above-depth, breadth, mechanisms and activities—they may be applied to any decision context to measure PDM. In order to capture the context for PDM, it is necessary to identify specific decisions of appropriate levels (strategic/tactical) and types (operations/marketing, core functions/support functions). When decision processes in one organization are the focus, actual decisions made in that organization can be used. Such an approach would provide an accurate, but idiosyncratic, measure of PDM within a specific organization. When cross-organizational comparisons are the purpose, which is most common in nursing systems research, a standard set of decisions must be used. In some studies, it may be sufficient to use decisions already identified in other studies such as [43] the four very general decisions named in Hage and Aiken's [44] PDM scale, or the decisions in Table 5. To enable cross-organization comparisons in this study, we identified the set of hypothetical, realistic, and easily recognized decisions through a modified Delphi as described above and shown in Table 5. Figure 3 shows the scoring instructions.

## 2. Methods

This study is a secondary analysis of data in two studies conducted at the University of Texas at Austin and approved by their Institutional Review Board. Duke University Medical Center Institutional Review Board approved the secondary analysis of data. Informed consent was obtained from all participants. We examined the PDM of members of two work groups, registered nurses (RNs), and certified nurse assistants (CNAs) in nursing homes using two different samples. Studying the PDM of more than one work group allowed us to test the PDMI's applicability to distinguish between the participation of workers in different job categories. RNs are clinical professionals with extensive education and socialization into a profession. CNAs, on the other hand, are low skilled workers with only about 6 weeks of training. These two groups possess very different capacities as sources and processors of information, providing a more challenging test of the validity and reliability of the PDM measure than if we studied only one target group.

We evaluated the PDMI in two studies. Below, we describe the sample and procedures for each study and then present the results.

### 2.1. Study One: Single Informant Observation

In Study I, we analyzed the items of the decision making measures to (1) assess reliability and validity and (2) reduce the number of decisions from the initial six (per decision type as depicted in Table 5) developed in the Delphi study, to a smaller number such that respondent burden is minimized but reliability and validity are maintained, and (3) examine the limits of the PDMI's usefulness across varying levels of occupational, technical, and professional groups by measuring the participation of two target groups, RNs and CNAs.

#### 2.1.1. Data Collection Procedures

We invited all directors of nursing and nursing home administrators in 589 nursing homes in Texas, which employed one or more full-time RNs, to serve as mail-survey informants and to personally complete and return-mail the questionnaires. Replacement materials were sent to nonrespondents after six weeks. For purposes of evaluating test-retest reliability, we mailed a second copy of the PDMI after six weeks to all who responded to the first mailing.

#### 2.1.2. Sample

Surveys from 238 informants were returned from 197 (34%) of the homes. We chose to use a single key informant and thus dropped 41 responses of nursing directors in nursing homes for which we also had an administrator response. These administrator and director pairs did not differ in their responses (*P* > .05 level). Informants from 116 (59%) homes completed a second copy of the PDMI for test retest. Most informants were administrators (58%) and females (83%). Tenure in their current position was 4.04 years (SD, 4.84), although 75% reported 7.47 (SD, 7.00) years of previous experience in a similar position. The nursing homes, on average, had 127 (SD, 51.5) licensed beds, employed 2.35 RNs (SD, 2.2), 2.76 licensed vocational nurses (SD, 6.6), and 38.45 CNAs (SD, 22.37), and were for profit (93%).

### 2.2. Study II: Multiple Informant Observation

In Study II, we wanted to strengthen the test of the PDMI by replicating Study I with an expanded key informant pool and thus included administrators, nursing directors, RNs, licensed vocational nurses (LVNs), and CNAs. The RNs, LVNs, and CNAs (personnel in the nursing department) were added as informants in Study II because as the largest group of workers in a nursing home, they are in a position to observe the decision patterns of RNs and CNAs in the organization. In addition, we improved the sampling procedures to overcome potential biases of the mail survey of Study I. The purposes of Study II were to (1) establish that the PDMI measures were meaningful at the organization level, (2) further assess reliability of the PDMI, (3) further examine the validity of the operationalization of the PDM model in terms of the decision context, and (4) assess construct validity.

#### 2.2.1. Data Collection Procedures

We used a proportional, stratified random sampling procedure to select a sample that represented the population distribution of profit and nonprofit nursing homes and to capture the geographic and racial diversity of Texas. The criterion for inclusion was nursing homes that had one or more RN FTE(s). We contacted 380 nursing homes to participate. A total of 195 (51%) nursing homes agreed to participate and 3903 individuals completed surveys. One of the investigators or research assistants visited each nursing home and collected data from staff that attended an education program that we provided.

#### 2.2.2. Sample

Of the 195 homes, 86% were for profit. The average size was 113 beds (SD, 53.54) with 84% occupancy. On average, the nursing homes employed 4.04 RNs (SD, 3.84), 9.22 LVNs (SD, 7.65), and 29 CNAs (SD, 19.08). Most directors of nursing (DONs) were female (91%) with an average of 2.6 years in their present position and 5.8 years of experience in a similar position. Most of the NHAs were also females (55%) and had been in their positions for an average of 5.3 years with 9.5 years of experience in a similar position. Most NHAs and DONs were white (85% and 80%, resp.) with black being the second most predominant for both NHAs and DONs (4.5% and 6.7%, resp.). The majority (66%) of NHAs held a Bachelor's degree or higher while only 32% of DONs held a Bachelor's degree or higher. Responding RNs, LVNs, and CNAs were also mostly female, but, as a group, they were more racially diverse than the NHAs and DONS. The CNAs, for example, reported being majority minority (35% Black; 22% Hispanic).

### 2.3. Instrumentation for Validity Testing

To assess convergent validity, we used a global participation measure, Hage and Aiken's [44] four-item, five-point scale, asking how frequently RNs and CNAs participate, in general, in decisions about hiring, promotion, new policies, and new programs. To assess discriminant validity we measured decentralization using Hage and Aiken's [44] 5-item, 4-point, Hierarchy of Authority scale and we measured formalization using Hage and Aiken's 12-item, 4-point, Formalization scale. Formalization is the degree to which work processes and procedures are codified and use of written rules and procedures is checked by managers [44]. Previous research demonstrated adequate validity and reliability for the measures of global participation, decentralization, and formalization in a variety of samples [45, 46], including nursing homes [23, 47, 48].

## 3. Results

### 3.1. Study I

*Data Reduction.* We conducted item analysis to identify poorly functioning PDMI items as a first pass at reducing the scale's length, which was initially 24 items (6 decisions × 4 items). We removed the decision (indicated by one asterisk in Table 5) that had the lowest item-to-total correlations for its associated PDMI items. This left for further analysis five decisions in each type for a total of 20 items on each PDMI scale. To further reduce the PDMI's length, we used principal components analysis using Varimax rotation [49] to identify the smallest number of decisions that maintained the theoretically expected structure of a Scope and Intensity component. The item scores for each type of decision were factor analyzed separately for each target group of RNs and CNAs. The result was 3 decisions and 12 items in each decision type. In Table 5, a double asterisk indicates the decisions eliminated through factor analysis.  Table 1 displays the means and standard deviations for all observations in Study I.

*Reliability.* Alpha coefficients were .85 or above for all PDMI measures (Table 1), indicating high internal consistency [49]. Stability was assessed using test-retest correlation coefficients over a six-week period. Test-retest coefficients were moderate, ranging from .40 to .64, suggesting that the construct may not be stable over time.

*Decision Context.* The mean scores (Table 1) for PDM differed by group (repeated measures MANOVA, *F* = 213.00, *P* < .0001, effect size = .417). Comparison of means showed that RN PDM scores were higher than CNA PDM scores (*P* < .0001). Repeated measures MANOVA also showed that mean PDM scores were higher for tactical than strategic decisions for RNs (*F* = 260.05, *P* < .0001, effect size = .546) and CNAs (*F* = 334.12, *P* < .0001, effect size = .607).

*Convergent and Discriminant Validity.* The results provide evidence of convergent and discriminant validity for the PDMI (Table 1). In this study, the moderate correlations among the PDMI scales and Hage & Aiken's global measure of participation are statistically significant (*P* < .01) suggesting convergence. The pattern of weak, or nonstatistically significant, correlations among the PDMI scales and the measures of decentralization and formalization suggest discriminant validity.

### 3.2. Study II

*Organization Level Properties.* To establish that the PDMI measures were meaningful at the organization level, we examined the ETA-squared coefficient (**η** ^*2*^), which estimates the proportion of variance in scores, that is, due to organizational membership and the intraclass coefficient or *ICC*(1, *k*), which estimates interrater reliability of aggregated items scores [50]. The ANOVA tests were all statistically significant indicating that the PDMI was tapping an organizational level construct. The **η** ^*2*^ ranged from .10 to .14 (Table 2). Although a minimum acceptable value for **η** ^*2*^ has not been documented, Joyce and Slocum [51] suggest that acceptable values may range up to .50, with a median of approximately .12. The values of *η* ^2^, in this study, indicated that the organizational membership accounted for at least 10% of variance in PDMI scores. The intraclass correlations, *ICC*(1, *k*), ranged from .52 to .66 (Table 2). These results suggest acceptable interrater reliability based on criteria from Kenny and Voie's [52], and we achieved greater values of *ICC*(1, *k*) than reported in previous studies [53]. Together the *ICC*(1, *k*) and *η* ^2^ coefficients provide confidence that these measures are meaningful as organizational-level variables. Therefore, we aggregated the item scores and total scores to the nursing home level, averaging across responses from all informants.

*Internal Consistency Reliability.* Alpha coefficients were .90 or above for all PDMI measures (Table 2), indicating high internal consistency.

*Decision Context.* The mean scores for PDM differed between RNs and CNAs (repeated measures MANOVA, *F* = 843.82, *P* < .0001, effect size = .946). Pairwise comparisons demonstrated that RN PDM was greater than CNA PDM for each of the four decision types (*P* < .0001). Repeated measures MANOVA showed that PDM scores were greater in tactical than in strategic decisions for RN PDM (*F* = 218.76, *P* < .0001, effect size = .694) and CNA PDM (*F* = 193.00, *P* < .0001, effect size = .651).

*Confirmatory Factor Analyses.* For each decision type (strategic operations, strategic marketing, tactical core functions, and tactical support functions), we used SAS PROC CALIS SAS Institute Inc. (2004) to perform a confirmatory factor analyses (CFA). The objective of these analyses was to test that for each decision type, and two underlying factors were needed to fit the 12 items dealing with depth, breadth, activities, and mechanisms respectively (i.e., to fit the conceptual model in Figure 1). We performed separate analyses for RN PDM and CNA PDM, resulting in a total of eight separate analyses (4 decision types × 2 target groups). Items dealing with depth and breadth were hypothesized to load on one factor (“Scope”), while items dealing with activities and mechanisms were hypothesized to load on a second factor (“Intensity”). These two factors were hypothesized to be correlated.

Our model building procedures are summarized in Table 3. Within each area of decision making, we first estimated a one-factor model (model b) and the two-factor Scope and Intensity model (c). Across decision making areas, the two-factor model provided a significantly better fit of the model to the data, as indicated by the CFA likelihood-based chi-square (model b versus model c) difference tests in Table 3. However, values of the Bentler and Bonett [54] normed fit index are well below the suggested cut-point of .9 [55] for an adequate fit of the model to the data. Following [55], we assessed model fit using multiple indices—Bentler and Bonett's [54] normed and nonnormed indices, and Bollen's [55] normed and nonnormed indices. Values of these other indices, which are presented for the final models in Table 4, were similar to those for the Bentler and Bonett normed index.

**Figure 2:**
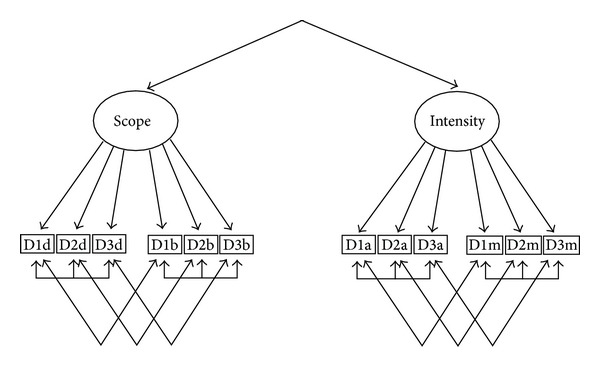
Model (d): the factor model that fits PDM data where D1d = decision 1 depth; D2d = decision 2 depth; D3d = decision 3 depth; D1b = decision 1 breadth; D2b = decision 2 breadth; D3b = decision 3 breadth; D1a = decision 1 activities; D2a = decision 2 activities; D3a = decision 3 activities; D1m = decision 1 mechanisms; D2m = decision 2 mechanisms; D3m = decision 3 mechanisms.

An examination of the model 1c residuals and modification indices (strategic operations decisions among registered nurses) revealed two patterns of method-related residual covariation. Items dealing with same decision (1 versus 2 versus 3; see Table 3) exhibited substantial residual covariation with one another, as did items with the same substantive focus (depth versus breadth versus activities versus mechanisms). This pattern of method-related covariation was not unexpected given similarities and differences in foci across items within hypothesized factors. Based on this pattern or residual covariation, we estimated model (d) (Table 3), which allowed for correlated errors among relevant indicators as indicated in Figure 2. This model had an adequate fit to the data, and we then replicated it in each area of decision making (i.e., strategic marketing, tactical core functions, and tactical support functions) for both the RN and CNA target groups (Table 3). All fit indices for model (d) exceeded .9. The Bentler and Bonett fit index measures the proportional reduction in the independence-model-based fit function (chi-square) associated with a given model and can be interpreted as the proportion of the total variation in the indicators explained by a given model. Comparing the values of this fit function in models (c) and (d) indicates that the two factors explain between 62% and 80% of this variation while the correlated errors explain another 17% to 30%. Finally, because our initial tests for two versus one factors did not include correlated measurement errors, we repeated this test with the correlated errors (Figure 2) included in the models compared. Again, the two-factor model provided a significantly better fit to the data in each area of decision making.

Standardized factor loadings, interfactor correlations, and measures of model fit for our eight final CFA models are given in Table 4. All factor loadings are statistically significant and range from .50 to .94 with most loadings exceeding .70. Interfactor correlations are also significant and range from .34 (3) to .88 (6). As noted above, the two-factor model provided a significantly better fit to the data despite the presence of high interfactor correlations in CNA PDM strategic marketing decisions, tactical functions, and tactical support decisions (6–8). In general, interfactor correlations appear to be considerably higher among CNA PDM compared to RN PDM. All but four (of 32 fit indices) are .9 or greater. Three of the four that are less than .9 are on normed fit indices, which can be deflated when the sample size is not large [55, ch. 7]. In general, the two-factor model provides an adequate fit to the data when patterns of correlated measurement error are taken into account. As observed in Table 4, the factor structure accounts for the majority (60–80%) of the variation in the observed indicators while correlated measurement error accounts for an additional 20–30%.

In Study II, we further examined the construct validity of the PDMI by assessing the extent to which the PDMI relates to measures of other organizational characteristics as theoretically expected (convergent and discriminant validity). To ensure the appropriateness of our test of hypotheses, we had to first establish that the measures of decentralization and formalization were meaningful at the organization level. Thus, we examined the **η** ^*2*^, *ICC*(1, *k*), and alpha coefficients. The ANOVA analyses were statistically significant for both variables and the *η* ^2^′s, *ICC*(1, *k*), and alpha coefficients of the aggregated items were adequate (Table 2).

The hypothesized relationships between the PDMI and a global measure of PDM and decentralization (H5) and formalization (H6) were supported (data not shown; see Supplementary Material Table 1 available online at http://dx.doi.org/10.1155/2013/706842). There were no statistically significant correlations between decentralization and PDM for tactical level decisions for either RN PDM or CNA PDM. The correlations between strategic level decisions and decentralization were weak but statistically significant (*P* < .05). There were no relationships between the RN PDM and formalization, which is the degree to which work processes and procedures are codified and enforced. For CNAs there were weak, significant relationships between formalization and PDM for strategic operations and marketing decisions.

## 4. Discussion

In this paper, we presented a multidimensional measure of participation in decision making (PDM) as a systems-level property of organizations, which we developed by drawing on complexity science, recognizing the complex adaptive nature of organizations. Although PDM has often been treated very simply as “shared influence” between superior and subordinate [27, 56], we argue that PDM is a more complicated construct when systems-level properties are recognized. In our model (Figure 1), we suggest two major components of PDM—Scope and Intensity. We envision Scope to capture the depth and breadth of participation by various agents, for example, including expertise of staff in a variety of roles such as nurse aides, housekeeping, and social work, and Intensity, for example, using a variety of ways of connecting people such as meetings and chance encounters in the hallway which might spawn spontaneous discussion and involving them in a variety of decision activities such as defining the problem or suggesting alternatives. It is one thing for a manager to bring together multiple agents, who represent different amounts of expertise [5]—*Scope*—it is quite another to allow those agents to interact at multiple points in the decision process, through multiple mechanisms—*Intensity* [11, 17, 57, 58]. Our Participation in decision-making instrument (PDMI) captures these aspects of PDM. Results of two studies demonstrated evidence of validity and reliability of the PDMI in two samples while measuring PDM of agents in two distinct job categories registered nurses (RNs) and certified nurse assistants (CNAs).

In order to assess and validate the PDMI, we tested it in a series of studies. We collected data from two separate nursing home samples. In Study I, we examined the psychometric properties of the PDMI using a single respondent in each of 197 organizations. In Study II, we examined the psychometric properties of the PDMI and examined the construct validity of the PDMI in a second sample of 195 organizations with 3,968 respondents. Rigorous methods used in Study II, including a random, proportionally stratified sampling procedure and the inclusion of multiple respondents from each nursing home who were in various job categories, improved the test of the PDMI, and increased our confidence in the reliability and validity of the measure of PDM. The informants responded to questions about PDM of two groups of workers that differed in terms of their education, their place in the hierarchy in the organization, and in their roles. Because RNs and CNAs possess very different capacities as sources and processors of information, measuring PDM of both groups provided a more challenging test of the validity and reliability of the PDMI.

*Reliability.* In both samples, the PDMI measures for all decision types (strategic operations and marketing; tactical core functions and support functions) demonstrated adequate internal consistency reliability of .86 or higher when measuring PDM of two distinct set of workers. In Study I, the PDMI scales demonstrated moderate stability, test-retest correlations of .40 to .64, over a period of six weeks. While we expected the test-retest correlations to be higher, it may well be that PDM; when measured as an emergent systems-level property of organizations, high levels of stability should not be expected. Because emerging properties are generated by interactions at the local level [17, 18], they are inherently dynamic. In terms of how PDM was measured—varying people of varying expertise and values interacting at the local level through a variety of means (e.g., chance encounters, informal meetings, and committee structures)—there is substantial room for variation, even over six weeks in time. What is not known from this study is to what extent PDM as a systems-level property will vary over time and how such variation relates to organizational performance such as patient safely, patient outcomes, and quality of care. We suspect that over the short term, PDM varies only moderately and as such is a fairly enduring characteristic of a particular organization—that is, it has sufficient stability to be meaningful in a cross-sectional study and as a management strategy. However, stability is an important question for future research.

*Decision Context.* In developing the PDM model using complexity theory, we proposed that PDM has systems-level properties that are ignored by prior measures. Modeling PDM as a systems-level phenomenon captures the richness of connections and interaction among agents within an organization [9]. In Study II, because we had multiple informants from each organization, we were able to test whether PDMI scores were meaningful when aggregated to the organization level and found that the eta-squared and *ICC*(1, *k*) coefficients supported this view.

We also argue that a model of PDM should include the decision context by varying the type of decision (i.e., strategic or tactical) being made. We believed that the PDMI would capture differences in PDM between agents with different expertise or roles and that the PDMI would capture differences in PDM by decision type—based on the expertise of the professional/technical agents involved. The data in both Study I and Study II demonstrated strong support for both of these arguments. Specifically, at the individual and organizational level, PDM differed between RN PDM scores and CNA PDM scores and PDM scores were higher for tactical than strategic decisions for RNs and CNAs. Therefore, we can draw the conclusion that PDMI is valid with respect to decision context (type of decision being made). In this paper, we provided details of the procedures used to customize the decision context for the nursing home settings. To use the instrument in other settings, researchers can follow similar steps to developing the decision context that is appropriate for the study setting. For example, this instrument has been used in hospitals to measure the PDM of physicians, nurses [29, 59] and mid-level managers [29], and the influence of nurse executives [60], in public health to measure the PDM of professional and nonprofessional case managers [58], nursing homes to study RN PDM [23, 61], and in nursing homes to study falls prevention [17]. In each study, different decision contexts (decision type, content, and agents participating) were developed.

*Validity.* Across the two samples, there is substantial support for the construct validity of the total PDMI scale scores. In Study I, convergent and discriminant validity was demonstrated for all PDMI scales providing evidence of validity consistent with our expectations. The moderate correlations with a global measure of PDM demonstrated that the scales tap the domain of PDM. The low-to-zero correlations with different but related dimensions of organizational structure (decentralization, formalization) demonstrate the PDMI's capacity to discriminate between constructs. In Study II, we found additional support when, in general, the PDMI scores were weakly or nonsignificantly correlated with decentralization and formalization. When measuring RN PDM, we found full support for the notion that PDMI would correlate positively and significantly with communication openness and communication accuracy. We did not find the same correlations for all of the measures of CNA PDM, however. When measuring CNA PDM, operations PDM did not correlate significantly with communication openness and support-functions PDM did not correlate with communication accuracy. This finding might be explained by the differences between professional and unlicensed staff role preparation which opens questions for future research.

**Figure 3:**
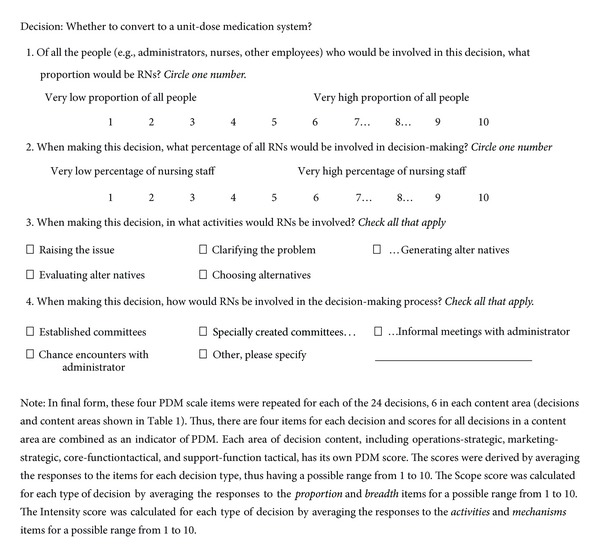


Confirmatory factor analysis of Study II data provided evidence of support for the notion that PDM has two dimensions and that a two-factor model will best fit the PDMI data. The PDMI measures for all four decision types and both target groups demonstrated evidence of the distinction between the underlying constructs of Scope and Intensity. The analysis also revealed, however, that the measure produced substantial method's variance related to the fact that the four PDMI items of depth, breadth, activities, and mechanisms were repeated across three decisions. Covariation related to the response patterns for each decision, however, was small in comparison with the variation accounted for by the construct dimensions of Scope and Intensity. This methods variation most likely stems from the way that the questionnaire presented the items (see Figure 3). At the top of each page was a different decision scenario, followed by the set of four PDMI items. This method's variance might be avoided with a different presentation of the items in the questionnaire. For example, the depth item could be presented followed by a list of decisions (repeat for each of the four items). Further testing of the PDMI is needed to address this issue. The fact that each decision has covariation associated with it is not particularly troublesome in the total score because we anticipated variation in PDM based on decision context.

## 5. Summary and Conclusions 

An important conclusion of this study is that participation in decision making (PDM), when modeled as a systems-level property of organization, has multiple dimensions and is more complex than it has been viewed in traditional measures of the construct. Depending on the decision context, managers can alter the nature of connections and interactions among agents in a system by accounting for the depth and breadth of expertise needed—*Scope*—and when and how agents connect to the decision process and to each other—*Intensity.* Prior studies have demonstrated that the effects of PDM vary by decision type [61, 62] and decision process [10] and there is no one optimal level of participation for all involved. Understanding the PDM components of Scope and Intensity expands the options available to managers who want to tap the talents and expertise of different organizational members in ways that will improve how information is processed and interpreted [11, 17]. The results of this study mean that PDM does not assume a single level across all decisions or groups, as if it were a stable characteristic of an organization or even a “management style” that is used to influence job satisfaction of workers [1, 5, 63]. These findings are important for future research on PDM because they suggest that an organization knowing how an organization involves is members is not a simple global phenomenon. PDM will vary with the type of decision and with the nature of the available participant. Thus to use a global measure of PDM does not capture PDM as a systems level property. It is important for managers and consultants to accept the notion that PDM is a multidimensional concept and cannot be managed with a broad brush, that is, guidelines such as “people should be allowed to participate in decisions that affect them.” This type of guideline would prevent managers from gaining the benefit that nursing staff can bring to decision making across a variety of strategic and tactical decisions, even those which may directly impact their work.

A second conclusion we draw from this is that unidimensional measures will not adequately account for the systems-level properties or complexity of PDM. Thus, when asking research questions, researchers need to clarify the theoretical foundations. If asking questions about PDM as a managerial strategy for improving the effectiveness of decisions, then the PDMI measure will provide a valid indicator for exploring relationships between PDM and performance. The PDMI is an organizational-level measure of the systems-level property, PDM. However, if the research interest is about individual-level phenomena, such as the relationship between individuals' perceptions of their own participation and how it relates to job outcomes or satisfaction, the PDMI is not an appropriate measure because it does not measure attitudes about PDM or individual-level behaviors.

We have offered both theoretical and empirical support for conceptualizing PDM as a complex construct with multiple components and a systems-level property of organizations. The value of this conceptualization for nursing managers is the new meaning it gives to PDM, the additional mechanisms such as chance encounters and informal meetings, that become apparent for making it happen, and the benefit it gives organizations operating in complex and unknowable worlds. The value of this conceptualization for researchers is that entire new research domains are illuminated. What components of PDM become more or less important as the organization is in the midst of change? Under what conditions does scope of PDM offer benefits to the manager that Intensity does not and vice versa? Moving toward a more complex view of PDM clarifies, how people come together in organizations to make decisions and interpret their worlds. When we conceptualize PDM as an emerging property of organizations, then the role of PDM in organizational functioning becomes clearer. We have attempted in this analysis to enhance our understanding of PDM when it is conceptualized as a complex systems-level phenomena.

## 6. Example of How Items Are Attached to a Decision in the Measure

For more details, see Figure 3.

## Supplementary Material

Supplementary Table 1. Study II: Validity Correlation Coefficients for Aggregated Total PDM Scores (N=195).

## Figures and Tables

**Table 1:** Study I: Single informant means, standard deviations, reliability coefficients, and convergent and discriminant validity coefficients.

Variable	M (*N* = 197)	SD	Alpha (*N* = 197)	Test-retest (*N* = 113)	Pearson correlations (*N* = 197)		
					Convergent validity	Discriminant validity	
					Global measure of PDM	Decentralization	Formalization
PDM—RN measures							
Operations	4.97	2.32	0.93	0.64**	0.569**	−0.040	0.028
Marketing	3.83	2.45	0.93	0.47**	0.448**	0.034	−0.057
Core functions	6.72	2.33	0.93	0.52**	0.565**	0.110	−0.161*
Support functions	5.96	2.29	0.93	0.61**	0.512**	0.138	−0.162*
PDM—CNA measures							
Operations	2.32	1.15	0.86	0.64**	0.268**	−0.076	0.080
Marketing	1.92	1.11	0.88	0.40**	0.267**	0.079	−0.117
Core functions	3.58	1.47	0.89	0.47**	0.405**	0.136	−0.088
Support functions	3.30	1.50	0.88	0.56**	0.286**	0.166*	−0.197**
Hage & Aiken Scales							
RN global participation	3.62	1.09	0.87	—	—	—	—
CNA global participation	2.37	0.76	0.73	—	—	—	—
Decentralization	3.01	0.73	0.87	—	—	—	—
Formalization	3.14	0.34	0.67	—	—	—	—

Note. *N* = 197. *N* = 113 for test-retest correlation coefficient.

**P ≤ 0.01.

*P ≤ 0.05.

**Table 2:** Study II: Organization means, standard deviations, and reliabilities of total PDM scores (*N* = 195).

Variable	M	SD	Eta-squared	*ICC*(1, *k*)	Alpha
PDM—RN measures					
Operations	5.58	1.04	0.14	0.66	0.94
Marketing	6.10	1.08	0.13	0.64	0.95
Core functions	7.03	0.74	0.10	0.52	0.90
Support functions	6.65	0.78	0.11	0.56	0.90
PDM—CNA measures					
Operations	2.57	0.67	0.13	0.64	0.95
Marketing	3.02	0.73	0.12	0.60	0.94
Core functions	3.75	0.72	0.10	0.52	0.93
Support functions	3.37	0.70	0.11	0.58	0.93
Additional instruments					
Decentralization	3.00	0.40	0.09	0.48	0.87
Formalization	3.80	0.22	0.11	0.57	0.79
Communication openness	3.55	0.33	0.14	0.65	0.89
Communication accuracy	2.89	0.26	0.09	0.45	0.81

**Table:** (a) Registered nurse participation in decision making

Model	Decision type			
	(1)	(2)	(3)	(4)
	Strategic operations	Strategic marketing	Tactical core functions	Tactical support functions
(a) Independence: chi-square/df	3095.9/66	3374.5/66	2708.2/66	2551.0/66
(b) One factor: chi-square/df/(*P*)	1409.7/54/(<0.001)	1413.0/54/(<0.001)	1519.2/54/(<0.001)	1494.5/54/(<0.001)
Bentler and Bonnet normed fit	0.54	0.58	0.31	0.41
(c) Two factors: chi-square/df/(*P*)	881.2/53/(<0.001)	798.5/53/(<0.001)	879.1/53/(<0.001)	965.0/53/(<0.001)
Bentler and Bonnet normed fit	0.72	0.76	0.68	0.62
Chi square difference (b) versus (c)	528.5/1/(<0.001)	614.5/1/(<0.001)	640.1/1/(<0.001)	529.5/1/(<0.001)
(d) Two factors + corr. error: chi-square/df/(*P*)	143.0/35/(<0.001)	164.5/35/(0.001)	133.8/35(0.001)	157.8/(<0.001)
Bentler and Bonnet normed fit	0.95	0.95	0.95	0.94
Chi square difference (1 versus 2 factor models with correlated error)	103.2/1/(<0.001)	129.4/1/(<0.001)	79.0/1/(<0.001)	82.8/1/(<0.001)

**Table:** (b) Certified nurse assistant participation in decision making

Model	Decision type			
	(5)	(6)	(7)	(8)
	Strategic operations	Strategic marketing	Tactical core functions	Tactical support functions
(a) Independence: chi-square/df	2859.6/66	2702.1/66	2347.9/66	2385.8/66
(b) One factor: chi-square/df/(*P*)	982.3/54/(<0.001)	1023.3/54/(<0.001)	1025.0/54/(<0.001)	1069.9/54/(<0.001)
Bentler and Bonnet normed fit	0.65	0.62	0.56	0.55
(c) Two factors: chi-square/df/(*P*)	567.7/53/(<0.001)	799.7/53/(<0.001)	793.0/53/(<0.001)	886.4/53/(<0.001)
Bentler and Bonnet normed fit	0.80	0.70	66	63
Chi square difference (b) versus (c)	405.6/1/(<0.001)	223.6/1/(<0.001)	232/1/(<0.001)	183.5/1/(<0.001)
(d) Two factors + corr. error: chi-square/df/(*P*)	74.8/35/(<0.001)	127.6/35/(0.001)	155.1/35(0.001)	176.2/(<0.001)
Bentler and Bonnet normed fit	0.97	95	93	0.93
Chi square difference (1 versus 2 factor models with correlated error)	104.1/1/(<0.001)	26.6/1/(<0.001)	24.31/1/(<0.001)	15.4/1/(<0.001)

**Table:** (a) Registered nurse participation in decision making

	Decision type							
	(1)		(2)		(3)		(4)	
	Strategic operations		Strategic marketing		Tactical core functions		Tactical support functions	
	Scope	Intensity	Scope	Intensity	Scope	Intensity	Scope	Intensity
Decision 1: depth	0.70		0.90		0.91		0.59	
Decision 2: depth	0.84		0.90		0.77		0.75	
Decision 3: depth	0.92		0.88		0.85		0.86	
Decision 1: breadth	0.74		0.88		0.90		0.67	
Decision 2: breadth	0.87		0.91		0.72		0.90	
Decision 3: breadth	0.94		0.90		0.87		0.95	
Decision 1: activities		0.56		0.82		0.81		0.67
Decision 2: activities		0.85		0.81		0.75		0.72
Decision 3: activities		0.86		0.81		0.83		0.76
Decision 1: mechanisms		0.67		0.82		0.51		0.58
Decision 2: mechanisms		0.72		0.84		0.67		0.67
Decision 3: mechanisms		0.76		0.87		0.69		0.81
Interfactor correlation:	0.75		0.71		0.34		0.40	
model chi-square/*P* (df = 35)	143.0/<0.001		164.5/0.001		133.8/0.001		157.8/<0.001	
*Fit indices *								
Bentler & Bonett normed index	0.95		0.95		0.95		0.94	
Bentler & Bonett nonnormed index	0.93		0.93		0.93		0.91	
Bollen normed index	0.91		0.91		0.95		0.88	
Bollen nonnormed index	0.96		0.96		0.96		0.95	

**Table:** (b) Certified nurse assistant participation in decision making

	Decision type							
	(5)		(6)		(7)		(8)	
	Strategic operations		Strategic marketing		Tactical core functions		Tactical support functions	
	Scope	Intensity	Scope	Intensity	Scope	Intensity	Scope	Intensity
Decision 1: depth	0.78		0.84		0.78		0.59	
Decision 2: depth	0.90		0.84		0.73		0.77	
Decision 3: depth	0.83		0.77		0.79		0.73	
Decision 1: breadth	0.80		0.84		0.74		0.64	
Decision 2: breadth	0.93		0.79		0.76		0.83	
Decision 3: breadth	0.87		0.75		0.79		0.77	
Decision 1: activities		0.74		0.81		0.75		0.69
Decision 2: activities		0.87		0.80		0.75		0.75
Decision 3: activities		0.85		0.83		0.73		0.84
Decision 1: mechanisms		0.79		0.86		0.73		0.70
Decision 2: mechanisms		0.88		0.77		0.73		0.78
Decision 3: mechanisms		0.88		0.73		0.75		0.76
Interfactor correlation:	0.79		88		0.83		0.87	
model chi-square/*P* (df = 35)	74.8/<0.001		127.6/0.001		155.1/0.001		176.2/0.001	
*Fit indices *								
Bentler & Bonett normed index	0.97		0.95		0.93		0.93	
Bentler & Bonett nonnormed index	0.97		0.93		0.90		0.89	
Bollen normed index	0.95		0.91		0.88		0.86	
Bollen nonnormed index	0.96		0.97		0.95		0.94	

^a^All factor loadings and interfactor correlations significant at *P* < .0001.

**Table 5:** Decisions included in the participation in decision-making instrument.

Strategic decisions	
Operations-strategic decisions: (i) **Whether to create an admission team whose function it is to do preadmission screening? (ii) Whether to charge patients for special equipment they require or use a flat fee for all patients? (iii)^ ∗∗^Whether to create a specialized rehabilitation team to provide rehabilitation services or utilize regular staff? (iv) Whether to hire a registered dietitian as food service supervisor? (v) Whether to alter the mix of Medicaid, Medicare, or private pay patients? (vi) *Whether to create our own PRN pool of nurses or use an outside agency?	Marketing-strategic decisions: (i) **Whether to offer an amenities package aimed at private pay patients? (ii) Whether to develop brochures for specialized markets such as people visiting physician's offices, hospitals, banks, and so forth? (iii) Whether to expand the traditional nursing homes services offered to include day-care, home health care, and/or community health promotion clinic? (iv) Whether to run an advertising campaign targeting special populations such as people with aids or multiple sclerosis, suggesting the use of the nursing home's specialized services? (v) **Whether to alter patient charges based on charges of competitors? (vi) *Whether to mail a quarterly informative newsletter to all referral sources?

Tactical decisions	

Core-function tactical decisions: (i) **Whether to convert to a unit-dose medication system? (ii) **Whether activity personnel or nursing staff will transport residents to scheduled activities? (iii) Whether to transfer a patient to another unit within the facility? (iv) Whether to increase or decrease the amount of patient care delegated to nursing assistants? (v) Whether to use mechanical patient restraints, chemical patient-restraints, or no patient restraints? (vi) *Which patient procedures (intravenous infusions, tube feedings, dressing changes, etc.) should be accomplished by which staff?	Support-function tactical decisions: (i) **What schedule should be employed for preventive maintenance of patient care equipment? (ii) Whether to change the charting system to include flow charts or check lists and more or less forms? (iii) What levels of stock are needed in the satellite supply areas? (iv) Whether to alter the procedure for incident reporting? (v) **When to schedule laundry-service hours? (vi) *When to schedule fire drills?

*Decision eliminated through item analysis.

**Decision eliminated through factor analysis.
