# Supplementary material for: Participation in Decision Making as a Property of Complex Adaptive Systems: Developing and Testing a Measure
Source: Nurs Res Pract. 2013 Nov 21;2013:706842. doi: 10.1155/2013/706842 (PMC3857844; doi:10.1155/2013/706842)
Supplement: Supplementary file 1 — Supplementary Table 1. Study II: Validity Correlation Coefficients for Aggregated Total PDM Scores (N=195). [file 706842.f1.doc]

Supplementary Table 1.

Study II: Validity Correlation Coefficients for Aggregated Total PDM Scores (N=195)

|  | Pearson Correlation Coefficients | | | |
| --- | --- | --- | --- | --- |
| Variable | Decentralization | Formalization | Communication Openness | Communication Accuracy |
| PDM - RN Measures: | | | | |
| Operations | ‑.188** | .100 | .191** | .263** |
| Marketing | ‑.080 | .089 | .178** | .211** |
| Core-Functions | .110 | .100 | .253** | .120* |
| Support-Functions | .117 | .088 | .267** | .197** |
| PDM - CNA Measures: | | | | |
| Operations | ‑.290** | .130* | .080 | .138* |
| Marketing | ‑.162* | .129* | .173** | .140* |
| Core-Functions | ‑.003 | .063 | .150* | .125* |
| Support-Functions | ‑.090 | .087 | .144* | .100 |

* p ≤ .05

** p ≤ .01
